# Mothers’ Involvement in Pediatric Postoperative Pain Care in a Tertiary Healthcare Setting in Saudi Arabia

**DOI:** 10.7759/cureus.34967

**Published:** 2023-02-14

**Authors:** Fatmah I Saigh, Zainab I Saigh

**Affiliations:** 1 Oncology and Palliative Care, King Abdullah Medical Complex, Jeddah, SAU; 2 Clinical Psychology, Mental Health Hospital in Jeddah, Jeddah, SAU

**Keywords:** saudi arabia, pain management, pediatric, pediatric health care, collaborative care, pain care, mother participation

## Abstract

Background

Many children feel moderate to intense pain in the hospital following surgery. Untreated pain can have deleterious physical and psychological effects. Mothers' involvement in child pain care and management postoperatively has been shown to be important in improving the outcomes and experiences of children, mothers, and health professionals.

Aims

To explore mothers' involvement in postoperative pain care and management of their children during hospitalization and following discharge and identify approaches to improve management and participation activities.

Methods

We used a qualitative single case study design with thematic analysis. The analysis included 20 mother-child dyads and 21 nurses, involving observation of participants and semi-structured interviews of mothers and nurses. The analysis also incorporated a review of documents from the pediatric surgical department (hospital policies and forms).

Findings

The following main themes and sub-themes were generated from the data: (i) provision of pain information (expected type, frequency, and duration of pain after surgery, pain intensity score, pain relief medication, and pain management methods), (ii) communication deficiency (language barrier and breakdown in communication between health professionals), (iii) emotional and physical support (family support, environmental comfort, and sleep and meal requirements), (iv) social and cultural influences (patriarchal society, cultural and religious beliefs, and work status), and (v) hospital facilities, provisions, and services (entertainment, follow-up programs, education courses on pain management for nurses, and materials and services).

Conclusions

The study examined mothers' participation in postoperative pain care and management in a cohort of children admitted to a tertiary care setting in Saudi Arabia, highlighting key factors that influence involvement and suggesting approaches for improving participation.

## Introduction

A number of studies have examined parental participation in child health care in various areas of the world, including the Arabian Gulf [1], Asia [2-4], Latin America [5], and Western countries [6-9]. There is a broad consensus that parental involvement in the health care of their children, including their presence at the hospital, whether through active participation or management, is advantageous for both parents and children in terms of reducing physical and emotional distress and increasing the security and happiness of the child [10,5].

More particularly, mothers have been found to have a central role in child healthcare, often being in a unique position as the primary caregiver to understand the physical and emotional needs of the child postoperatively [5,11,12]. However, an emerging picture is that while mothers often engage in successful participation and management during hospitalization and at home, these can be met with a number of challenges, including a lack of awareness of their role, limited time in their busy schedules to be actively involved, not having support for non-hospitalized or healthy siblings, being subject to various societal, cultural, or economic pressures, not having basic physical and emotional needs met, and a lack familial support [13,10,4].

Children experience differential severity of pain following surgery [14,9]. If unacknowledged and untreated, postoperative pain in children can lead to possible complications, such as adverse psychological issues owing to trauma or even suppressed immunity and respiratory functions [15-17]. Czarnecki et al. posit that in view of the current high level of sophistication of global healthcare, pain assessment and management in children should be at a consistently high level [18]; yet many studies show that standards often do not meet broader expectations [19]. In a review article, Dorkham et al. examined the management and administration of treatment criteria for children postoperatively, finding that poor communication with health professionals, lack of ability to recognize and assess child pain, misconceptions relating to analgesics, and difficulty in obtaining appropriate medication were salient themes [20].

Mothers' involvement in pain care and management varies throughout the world. For example, Chng et al., in a study carried out in China, found that mothers display moderate levels of knowledge, attitudes, and incorporation of pain relief methods in postoperative pain care and management [2]. By contrast, Smyth et al. [21] and Ekim and Ocakci [22], in studies carried out in Australia and Turkey, respectively, observed that mothers displayed inadequate knowledge of child pain-relief methods. Overall, in the majority of studies reviewed it has been noted that mothers and health professionals feel the involvement of mothers is suboptimal and could be improved [23-25,4]. However, there are often limited resources, education, and guidance, which impact involvement, and can lead to a lack of understanding and confusion for both health professionals and mothers about their respective functions and responsibilities [3].

In Saudi Arabia, there is currently scant data available in studies, government documentation, case reports, and reviews on the current picture of mothers' involvement. To the best of the authors' knowledge, there is just one recent study examining family-centered care in a tertiary hospital setting that explored nurses' perceptions of the involvement of family members in Saudi Arabian hospitals [1]. However, since families were the units of study and nurses' experiences were the data source, maternal participation was not given sufficient focus to draw generalizations regarding involvement in child pain care. Nevertheless, it was found that family-centered care is becoming increasingly more accepted into practice by health professionals in Saudi Arabia, and perhaps by extension, across Arab Gulf states. It seems likely that the use of family-centered care strategies will be increasingly absorbed into the fabric of Arabian Gulf healthcare practices, which are traditionally conservative cultures that have undergone rapid social, technological, and industrial change in recent years.

There is a need for a greater understanding of mothers' involvement in postoperative pain care and pain management in Saudi Arabia, at the hospital and following discharge, to determine facilitators of and barriers to involvement toward improving practices, experiences, and outcomes. This study explores mothers' involvement in a tertiary hospital, Abdulaziz University Hospital, Jeddah, Saudi Arabia. The findings may benefit mothers through understanding deficits in childcare practice, and benefit healthcare professionals and healthcare systems to improve experiences and outcomes in the Gulf countries and beyond.

## Materials and methods

Design

This was a qualitative single case study with thematic analysis using mother-child dyads, nurse participants, and hospital documents. A non-probability purposive sampling technique was used, which is a technique in qualitative research, where the criteria used for participant selection is specific and the aim is to provide an in-depth understanding of the unit of analysis (mothers) through the actions and understandings of the people and institutions involved. The analysis comprised participant observation, semi-structured interviews of mothers and nurses, and a review of pediatric department hospital policies and forms. A mother and nurse interview guide was used, which is described below in the Interviews subsection. For observation, the researcher assumed an observer-as-participant stance, aiming for minimal involvement. The documents reviewed were hospital policy and procedure papers on postoperative pain management, patient and family educational provision, and patient discharge protocols, enabling researcher observation and participant interview transcripts to be contextualized. The thematic analysis involved the standard schematic steps: initial familiarization with the data, generating codes and themes, reviewing themes, and producing a written analysis.

Selection of participants

Using a purposive sampling technique, the number of mother and nurse participants selected was dictated by data saturation point. Following every observation and interview with mothers and their corresponding nurse participants, an analysis was carried out to determine whether saturation had been reached. Participant mothers were codified using the letter M and a unique integer (e.g., M1), enabling confidentiality. Similarly, participant nurses were identified using the letter N and a unique integer (e.g., N6), except the head nurse (HN). Data saturation was reached on M16, although the researchers added M17-M20 to confirm, and data saturation was attained on N17, although the researchers included N18-N21 to confirm. Thus, the final number of participants included for both observation and interview was 20 mother-child dyads and 21 nurses. The inclusion and exclusion criteria for mother-child dyads and nurse participants are shown in Table 1 and Table 2, respectively. Mother-child dyad and nurse demographic information is shown in Table 3 and Table 4, respectively.

**Table 1:** Mother-child dyad inclusion and exclusion criteria.

Inclusion criteria	Exclusion criteria
1. Mothers with children aged 3-14 years. (This age range can reliably report pain to mothers.)	1. Mothers of children with critical illness or severe disability. (These require unique care.)
2. Mothers with the main responsibility for guardianship and care postoperatively.	2. Mothers of adopted children. (Adoption may create instability in the mother-child relationship, and possibly render data incongruent or inadmissible.)
3. Mothers with children admitted not less than 24 hours following surgery. (Hospital protocol required that the patient remains at the surgical ward ≥ 24 hours following elective surgery. This also allowed mothers to become accustomed to the environment.)	3. Non-Arabic speaking mothers. (Mothers that do not speak the standard Arabic language may not be culturally assimilated.)
	4. Mothers that expressed a desire not to participate.

**Table 2:** Nurse inclusion and exclusion criteria.

Inclusion criteria	Exclusion criteria
1. Expressed interest to be involved in the study.	1. Registered nurses unable to give bedside care (e.g., nurse educators).
2. > 1 year experience in a pediatric surgical department.	
3. Valid registered licence to practice as a nurse.	

**Table 3:** Mother and child demographic information. SA: Saudi Arabian

Participant code	Age (years)	Nationality	Education level	Number of children	Working Status	Child's age (years)	Child's gender
M1	47	SA	Bachelor	4	Yes	3	Boy
M2	38	Non-SA (Ethiopian)	Bachelor	5	No	7	Boy
M3	35	SA	Diploma	3	No	4	Boy
M4	32	SA	Bachelor	4	No	6	Boy
M5	32	SA	Bachelor	1	Yes	4	Boy
M6	30	SA	Diploma	5	No	10	Boy
M7	39	Non-SA (Yemeni)	Diploma	2	No	10	Boy
M8	48	SA	Bachelor	7	Yes	10	Boy
M9	23	SA	Diploma	4	Yes	4	Boy
M10	32	SA	Diploma	1	Yes	12	Girl
M11	28	Non-SA (Yemeni)	Diploma	3	No	3	Girl
M12	25	SA	Diploma	3	No	3	Boy
M13	31	SA	Bachelor	5	No	7	Boy
M14	33	Non-SA (Palestinian)	Diploma	1	No	4	Girl
M15	28	SA	Bachelor	3	No	3	Boy
M16	35	Non-SA (Yemeni)	Diploma	3	No	5	Girl
M17	30	SA	Diploma	7	No	8	Boy
M18	40	SA	Diploma	6	Yes	12	Girl
M19	23	Non-SA (Kazakhstani)	Diploma	5	Yes	7	Girl
M20	28	Non-SA (Yemeni)	Diploma	2	NO	7	Boy

**Table 4:** Nurse demographic information. SA: Saudi Arabian

Participant code	Gender	Age (years)	Nationality	Education	Number of children	Number of experience years
HN	Female	35	SA	Bachelor	0	8
N1	Female	44	Non-SA	Bachelor	2	1
N2	Female	30	Non-SA	Bachelor	1	5
N3	Female	47	Non-SA	Bachelor	4	31
N4	Female	39	Non-SA	Diploma	3	10
N5	Female	38	Non-SA	Diploma	2	10
N6	Female	38	Non-SA	Diploma	0	12
N7	Female	40	Non-SA	Bachelor	2	12
N8	Female	41	Non-SA	Diploma	2	14
N9	Female	23	SA	Diploma	0	1
N10	Female	54	Non-SA	Diploma	3	20
N11	Female	42	Non-SA	Diploma	3	11
N12	Female	41	Non-SA	Diploma	4	8
N13	Female	25	SA	Bachelor	0	2
N14	Female	27	Non-SA	Diploma	0	3
N15	Female	40	Non-SA	Bachelor	2	20
N16	Female	34	Non-SA	Diploma	1	10
N17	Female	33	Non-SA	Diploma	2	10
N18	Female	40	Non-SA	Diploma	2	11
N19	Female	46	Non-SA	Diploma	2	11
N20	Female	50	Non-SA	Diploma	2	20

Data collection

**Figure 1:**
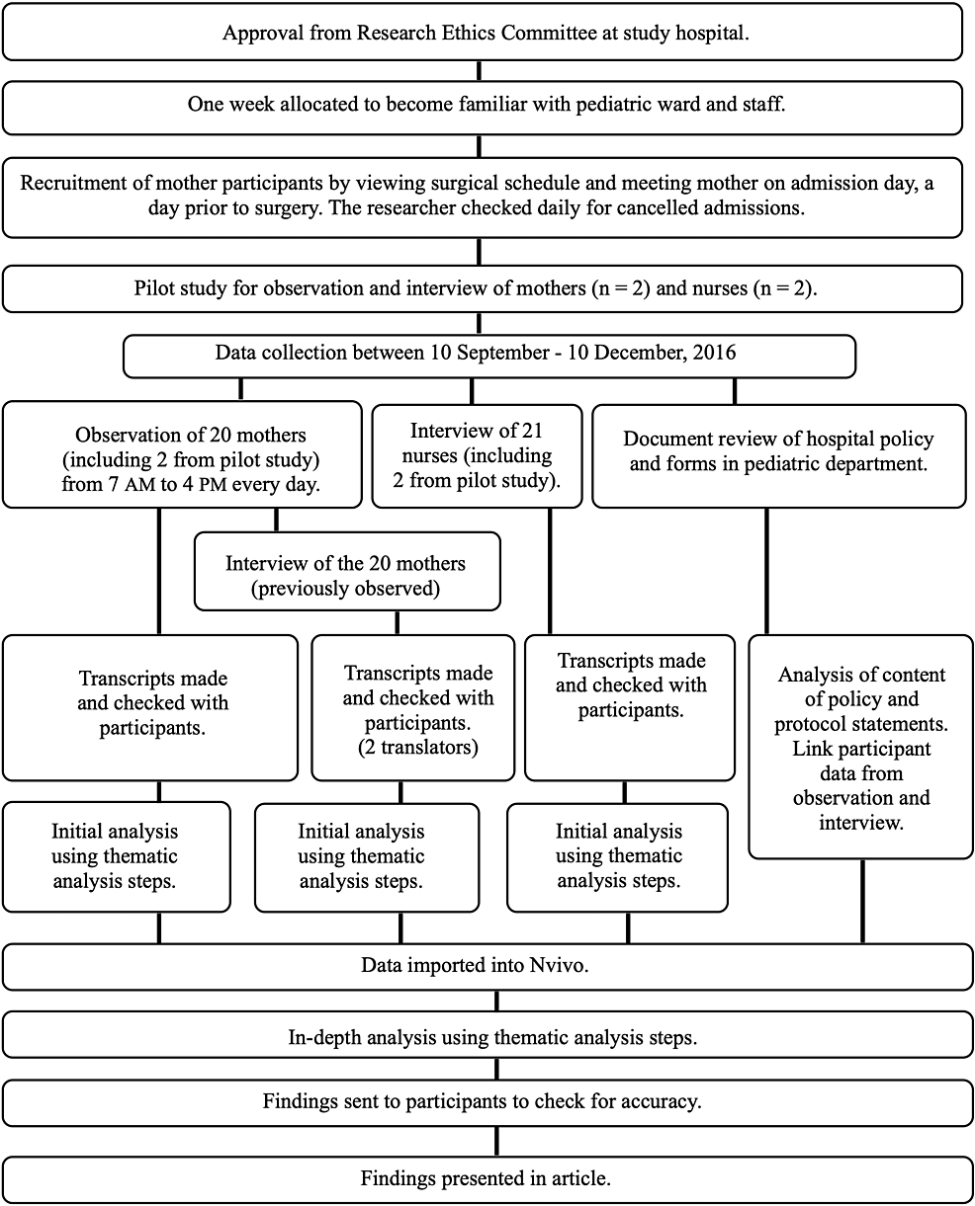
Flow diagram of study design.

The data were collected at King Abdulaziz University Hospital, Jeddah, Saudi Arabia, over a three-month period, from September 10, 2016, to December 10, 2016. The study setting was the hospital's pediatric surgical department, which has a complex, interconnected modern design. The pediatric surgical department included 52 beds, with 40 in use during the study period. There were 47 pediatric nurses employed at the department at the time of the study, with approximately 10-15 nurses on duty at any one time. Prior to observation, a pilot study was conducted on two mothers and two nurses, the data of which were included in the present analysis. A flow diagram of the study design is shown in Figure 1.

Observation

The internal hospital computer system was used to recruit mothers using a search for elective surgery within three months of the study commencement date. Mother participants were approached, and following given consent, but prior to observation, were asked to complete a participant demographics form. The form involved questions relating to the mother's age, number of children, education level, employment status, number of working hours per day, nationality, support sources, and the number of hospital admissions of her children and the child undergoing surgery, as well as the hospitalized child’s age, gender, and operation type.

It was noted that hospital family visiting times were not convenient or appropriate for carrying out mother observation because this was a busy period for both mothers and nurses. Also, it was not possible to observe mothers manage their children’s pain at home because in Saudi Arabian culture, it is not received etiquette to invite medical practitioners or other professionals into a domestic environment unless immediate and necessary healthcare is required [26]. One of the researchers observed mother-child dyads immediately following surgery in the recovery unit for approximately 30-60 minutes. It is hospital policy that mothers remain with children until they regain consciousness. Subsequent to this stage, children were transferred to the pediatric surgical department. Hospital policy requires nurses to check vital signs and measure pain over a period of two hours following surgery. In the pediatric surgical department, the researcher stayed in the nursing station and accompanied the assigned nurse. In general, children stayed in the department for a minimum of two days following surgery. The same researcher visited the children’s rooms in the department to conduct a series of observations during day shifts (7.00 AM - 4.00 PM). The final stage of care was the discharge of the child. Early in the morning on the day of discharge, the assigned nurse was consulted to see the exact time of discharge to be present for observation.

Interviews

Following written consent, convenient times and dates for mother and nurse interviews were arranged. These generally took place within a few weeks following the child’s surgery and hospitalization, usually during follow-up appointments. The interview guide incorporated a semi-structured interview technique, with open questions designed to let mothers and nurses freely explain their experiences. The mother interview guide involved four open questions on child care: the first was of a general nature, regarding the health condition for which the child was undergoing treatment; the second involved the mother's experience of surgery admission; the third concerned the mother's needs regarding care; and the fourth explored the mother's experience at home following discharge. The nurse interview guide consisted of three topics regarding healthcare management and mother-child care: the first concerned nurse perception of the management of children by mothers; the second explored how nurses prepared the child for discharge; and the third examined nurses' perception and understanding of mothers' involvement. The mothers' interview transcripts were translated from Arabic into English by a professional United Kingdom native translator. After the first translation, a Saudi-native translator translated the English transcripts back into Arabic to ensure the accuracy of the originals.

Data recording and analysis

Observation, interview, and hospital policy and form data were entered in tabular form in Word (Office 2011 for Mac; Microsoft Corporation, Redmond, Washington, United States) and subsequently arranged in a thematic form in NVivo version 11 for Mac, 2015 (QSR International LLC, Burlington, Massachusetts, United States). 

## Results

A number of main themes and sub-themes were highlighted by the researchers from observation notes, interview transcripts, and hospital documents. These are described in this section and supplemented with verbatim quotations from mother and nurse participants and hospital documents. The main themes are the following: provision of pain information, communication deficiency, emotional and physical support, social and cultural influences, and hospital facilities, provisions, and services.

Provision of pain information

Expected Type, Frequency, and Duration of Pain after Surgery

Providing adequate information regarding pain frequency and duration to parents following surgery is considered required knowledge in the hospital policy documentation. In general, mothers said they wanted to have an active role in pain management, to support the child. Mothers and nurses said mothers needed to know what to expect in terms of the frequency and duration of pain to properly prepare themselves and their child for the experience. Mothers and nurses noted that mothers desired to understand what procedures or treatment their child may be subjected to following surgery that may cause pain physically or emotionally, such as the insertion of an IV cannula or catheter. However, from the data, mothers and nurses voiced concerns regarding a consistent lack of preparatory information on the expected experience and duration of pain following surgery. The following quote from the interview with N9 illustrates these issues.

The mothers need information from doctors prior to surgery. The doctors should discuss the discharge care plan with the child’s parents and explain everything related to the surgery, including the procedure, pain relief goal, what device or line will be attached, and the pain relief methods. We (nurses) only follow the doctor’s instructions. (Interview N9)

Pain Intensity Score and Pain Relief Medication

Mothers often expressed a need to be involved in initiating their child’s comfort targets and goals. It was observed that when M12 was reunited with her child in the recovery unit, the on-duty nurse measured the vital signs (including pain score) but did not provide information in verbal or written form to M12 regarding pain care or management. A researcher regularly observed the facial expression of M12 during the first 10 minutes in the recovery unit, which often seemed to show agitation and anxiety. Shortly after this initial period, the researcher saw M12 crying and asking the nurse to give the child pain-relief medication. Indeed, M12 later asked the nurse why the child needed to be attached to a monitor with wires, and why the child had an oxygen mask. The nurse on duty only replied that the child was ‘okay’ and continued writing in the observation notes of the child’s medical file. While M12 was clearly worried that the child was experiencing severe pain, it was also apparent that she had a lack of understanding of hospital procedures, treatment programs, child pain experience and severity, knowledge of pain management and setting pain targets, and her particular role and duties in managing pain. In general, the researchers found that there was a lack of information given to mothers regarding child pain intensity score and pain-relief medication, although hospital pain management policy explicitly requires physicians and nurses to involve guardians in determining pain scales, as noted in the hospital documentation.

The physician and/or the nurse shall collaborate with the patient or guardian (the mothers) to determine the rating on the pain scale/sedation scale at which the patient would be able to function or have an acceptable quality of life. (Pain management policy, code: CLI-NU-084)

Pain Management Methods

According to hospital policy, mothers should be given preparatory information about how they can manage their child’s pain at the hospital and at home, following surgery. The hospital policy requires physicians or nurses to provide instructions to parents and children regarding pain relief methods, administration of medication, positioning of the child, child nutrition, and follow-up clinic appointments. From observation and interview, the researchers found that nurses did not give information routinely regarding pain management methods, and gave limited instructions on relevant factors for care, on admission day and during the hospitalisation period, as discussed in the following quote from the interview with M10.

I did not know how to relieve my child’s pain after surgery and I tried my best from my own understanding. My child was given medication for pain but she was still irritated because of surgery, and was not feeling comfortable. At that time, I felt I needed more information about how I can manage my child’s pain physically and psychologically. (Interview M10)

Communication deficiency

Language Barrier

Many mothers appeared to have difficulty in understanding nurses' instructions, or expressed problems related to understanding nurses during their interviews. This communication deficiency was often a result of a language barrier between native mothers, who spoke Arabic, and expatriate health professionals, who were required by hospital policy to speak English but generally spoke little Arabic, as shown in the following quote from the interview with M8.

I don’t speak English, but I can understand when the nurse uses a few Arabic wards and body movements, such as actions with her hands. (Interview M8)

An example of the language barrier was seen during the observation of M11. When the child of M11 woke up and screamed in the recovery unit, M11 asked the nurse in Arabic if she could put the child on her lap to make her feel more relaxed. The nurse consented to this in English, and her reply was intermixed with key Arabic words. However, the facial expression of M11 showed confusion, and the researcher observed that the mother subsequently did not put the child on her lap, which highlights that a lack of a common language, or lingua franca, is a significant problem for effective pain care communication.

Breakdown in Communication Between Health Professionals

A breakdown in communication between health professionals was found to limit the effectiveness of mothers' involvement in pain care. During her interview, M5 said that a nutritionist brought food to the nursing department when the child was fasting, prior to surgery. However, the nutritionist had not been informed of the fasting requirement. Her child subsequently cried when he realised he could not eat. This fuelled tension between mother and child, resulting ultimately in the child screaming and crying until surgery commenced. The following quote taken from the interview with N5 notes the importance of prioritizing up-to-date documentation to aid effective communication.

Documentation should be better than what we have in the ward. The nurse in the next shift needs to be updated about the patient’s pain care. (Interview N5)

Emotional and physical support

Family Support

Some mothers said in their interviews that they felt the need for greater family support at the hospital. In the study setting, the visiting times were 4:00 PM - 8:00 PM, and children were permitted to visit only on Fridays, which is a non-working day in Saudi Arabia. A number of mothers felt that their hospitalized child felt bored or tired, and would have benefited from increased visitation by family, particularly from children of a similar age, as suggested in the interview with M3.

The visiting times are not suitable for us. The child needs to enjoy time with the family, and cousins in particular. My child was sad because he could only see his twin at hospital. I stayed 15 days and we often felt bored. (Interview M3)

Environmental Comfort

Mothers generally did not feel comfortable sharing a room with other families in the nursing department.

I did not feel comfortable after the child had surgery because the room was not a private room (it was a two-bed room). (Interview, M3)

Mothers seemed to show frustration or agitation that they did not have privacy or a quiet environment for their child to rest, and often said that they were shy or embarrassed about the perceived noise or activity of their own children. During observation of M3, it was noted that her child cried and screamed in the first few days after surgery. M3 said during observation that she felt worried and uncomfortable because there was another child patient in the next bed who may have been disturbed by the noise. She said that she blamed herself also because her actions might have annoyed or disturbed the other child, and it was noted during observation that this self-consciousness seemed to limit her sense of confidence in dealing with her child’s pain, such as negatively impacting her ability to use distraction methods and constraining the quality of communication with her child and health professionals.

Sleep and Meal Requirements

It was observed that mothers were often very tired or had low energy prior to surgery, often because of inadequate sleep or food, which affected their ability to be involved in pain care. Mothers did not have allocated sleeping time, or were too busy to allocate sleeping time, because they were caring for their child. The following quote from observation of M13 highlights some causes of inadequate sleep.

I feel unwell because I did not sleep yesterday. I’m used to sleeping at home; this place is new for me. I have difficulty sleeping in new places, especially when the nurses come in to check my child or the other child. (Observation M13)

Mothers were also hungry because they were either too busy to prepare food, were not at home to have access to provisions, had limited access to hospital food, did not eat hospital food out of a lack of desire, or abstained from eating because their child was fasting before surgery.

Social and cultural influences

Patriarchal Society

While the social and cultural environment of Saudi Arabia is rapidly changing toward a more liberal worldview, and transitioning out of a traditionally patriarchal society, men still have a decisive influence on mothers' involvement in care. Accordingly, when dealing with the pain care and management of their child, some mothers preferred the presence of fathers or male relatives, and were apprehensive about making decisions without their presence, which impacted their confidence in being involved in pain care.

His (the child’s) father is responsible for organizing the surgery and finishing the paperwork (for admission and discharge). He tells me what should I do. In my culture, the man does everything and always talks with other people. (Interview M2)

Cultural and Religious Beliefs

The predominant religious and spiritual faith of Saudi Arabia is Islam, and mothers held a religious conviction that God protects their child. Mothers often prayed to God to ask for protection against any pain, complications, or possible death during their child’s surgery. It was found that some cultural and religious beliefs affected attitudes, understanding, and approaches, which were thought to limit successful pain care and management. For instance, N15 notes that religious ethics regarding food waste infringed on food safety.

Some mothers want to store the food (that we give them). But we tell them that if they do not eat it immediately they should throw it away. They often reply that this is ‘haram’ (which means it is an action against religious principles). (N15)

Thus, in the attempts made by mothers to adhere to cultural and religious principles, nurses thought that they may inadvertently be putting themselves and their child in danger, and thus lowering their ability to be effectively involved in pain care.

Work Status

The researchers noted that mothers seemed to have difficulty being involved in their child’s pain care owing to occupational commitments and work status. A number of mothers said that they could not arrange to take time off work to be actively involved in pain care. The following quote from the interview with M8 highlights this point.

I could not be with my child at all times when she was at hospital. I work as a teacher and now it is the end of term. During this period, it’s hard to take time off. (Interview M8)

Hospital facilities, provision, and services

Entertainment

Mothers and nurses often expressed a desire to have more variety and better quality entertainment at the hospital, which could help distract the child and support mothers in being more effectively involved in pain care. For example, in their interview, N1 made the following point.

I think they need to have more toys to divert the child’s attention. The child will forget his or her pain if such distractions are used. (Interview N1)

Follow-Up Programs

Mothers and nurses said that mothers required more integrated and comprehensive follow-up programs to support them following discharge. These were suggested to empower mothers with information about pain care so that they could feel more confident about being involved. Such information advised them about possible complications after surgery, including details of the specific outcomes related to their child, as noted in the interview with M1.

The hospital should follow up on the child’s condition even when the child is discharged. The doctors should consider the child’s circumstances individually. Some children might have other underlying health issues. They should put this in the treatment plan and provide extra support for the mothers after discharge. (Interview M1)

Education Courses on Pain Management for Nurses

Both mothers and nurses suggested that more comprehensive educational courses are required for nurses to better engage mothers in child pain care and to understand how they can more effectively interact with mothers and children. Mothers pointed out that nurses require education on how to tutor mothers on managing their child’s pain, as M16 discussed in her interview.

I felt nurses do not know how to deal with children. Some of them are tough. They treat the child as an adult. Others ignored the mother and expected her to know what she should do (regarding pain management) without telling her. (Interview M16)

Materials and Services

Nurses and mothers said that a greater quantity and better quality of materials and services are needed to improve pain care and management by mothers. Both mothers and nurses suggested that mothers required psychological support mechanisms to deal with the adverse behaviour of the child following surgery. Some nurses recommended extra support services, such as a dedicated pain management team, to advise and treat children for pain. This team could be standardized in the nursing department and involve routine or specialized visits that focused on pain. Mothers suggested the provision of group discussion sessions for mothers and health professionals to share knowledge to improve practice, as noted in the interview with M3.

Sometimes I went to talk with other mothers when I was at hospital. We had a chat and supported each other. I feel this made me understand what other mothers faced, and gave me tolerance about my own situation. (Interview M3)

## Discussion

An analysis of the data has enabled a comprehensive picture to be constructed regarding mothers' participation in postoperative pain care and management in this cohort in Saudi Arabia. Five salient main themes were found which are now discussed in the context of the wider healthcare literature. The findings may be used to inform future planning, development, and growth in healthcare systems and improve mothers' participation in Saudi Arabia and beyond. 

Provision of information on pain

The first theme, provision of information on pain, relates to the lack of information given to mothers about their role in their child’s pain care and management. Limited information may be explained as the product of the actions and attitudes of health professionals; often nurses and doctors give low priority to involving parents in pain management because they do not understand the significance of their role [1,27,18]. In the present study, mothers were found to have little understanding of the pain their child may experience, and often misunderstood instructions, misused medication, or had erroneous beliefs surrounding pain and analgesics, corresponding with the findings of some other studies [28-33]. Mothers generally depended on nurses for information on pain management, and thus required sound knowledge regarding practice, such as setting pain scores and use of analgesics. It was sometimes found that nurses themselves had poor knowledge regarding pain score setting, understanding of medication types, and use of analgesics, as found in a number of other reports [21,22,34-36].

Mothers were observed to be very anxious regarding their perceived ability to help their child deal with pain, physically and emotionally. At the same time, sick or hospitalized children were found to need greater emotional support than usual, owing to their inherent fear and anxiety of sickness, surgery, and hospitalization. Enabling mothers to be free from stress enables better performance in pain care, and mitigates children’s fear of pain and negative experiences [37]. Mothers and nurses recommended nursing education programs to further the understanding of pain care settings and the use of analgesics. Broadly, pain score setting, medication knowledge, and analgesics use were some main areas of weakness that need addressing by management bodies and health systems in Saudi Arabia to improve practice overall.

Communication deficiency

Communication deficiency is a theme that directly affects mother participation in pain care and management. Communication issues can result in negative attitudes and distrust in health professionals, and thus can limit involvement [38]. Comprehensive and meaningful communication between parents and health professionals has been found to be central to effective parental involvement in child care [5,39]. In the present study, communication problems were found to be mainly a product of a language barrier and lack of cultural understanding. These findings are supported by the results of a study conducted in Saudi Arabia by Almutairi on language barriers between health professionals, where it was observed that issues related to ineffective communication between patients, families, and healthcare providers were detrimental to quality health care [40]. Almutairi identified the language barrier and nurses’ lack of cultural understanding as key obstacles to quality health care. Also, in a study based in Italy, it was observed that a language barrier directly limits parental understanding regarding analgesics usage, and that even variations in the accent and dialect of nurses and parents can have adverse effects on the error rate of pain-relief medication [16].

In the present cohort, nurses responded that they often did not have enough time to have effective verbal engagement with mothers, owing to busy schedules, and thus were unable to successfully promote mother involvement. Ineffective nurse-physician communication has been found to be detrimental to patient safety, lower the quality of pain care, and increase costs [41,42]. In the present study, communication between health professionals was found to be ineffective and often error-prone, which corresponds with the findings of Czarnecki et al. [27] and Czarnecki et al. [18]. Mothers recommended that educational materials and courses be given to health professionals to improve communication skills and make them more effective in advising on pain care. This finding corresponds with that of Kodjebacheva et al., who recommend that health professionals undergo intervention strategies to improve communication [43].

Emotional and physical support

The theme of emotional and physical support relates to the optimal functioning of mothers to deal with pain care. The key aspects of physical support are a comfortable environment, adequate sleep, and good nutrition. Perhaps surprisingly, a hospital environment is sometimes considered a challenging place for mothers and children [44]. In the present cohort, mothers generally experienced little familial support at the hospital and following discharge. Also, mothers, who were generally the main caregivers of their hospitalized child, were often fatigued or lacking nutritional sustenance. These deficiencies negatively affected their ability to be actively involved in pain care. Regarding emotional support, it was found that visiting times were inconvenient for many extended family members, and children were not permitted to visit during standard visiting hours, limiting social relief for the hospitalized children. Indeed, playing with other children or family members can distract children from pain and suffering.

Shulkin et al. suggest that open visitation is a useful method to improve visitor experience and patient and family experience, and is thus welcomed by many nurses to enable effective collaborative care [45]. Indeed, in the present study, many mothers did not feel comfortable in their room in the nursing department. Limitations on privacy was considered by mothers to be one of the main causes of physical and emotional discomfort. Mothers expressed in interviews and during observation that they desired better surroundings and facilities to enable themselves to be more actively involved. Recent recommendations in the UK, for example, require that hospital rooms be comfortable, include retractable blinds or curtains, variable temperature systems, a welcoming ambience, interesting colours, natural daylight, a view of green vegetation, relative privacy, accessibility, and visual art that has universal appeal [46]. However, hospital environments can be challenging all over the world, and thus tertiary care facilitates in general require improvement to enable greater comfort for mothers, and accordingly perhaps improve involvement. Also, mothers were tired and hungry before surgery owing to a lack of sleep and food. Most mothers spent many hours beside their child to monitor them and provide companionship, but this also limited effective involvement in pain care. Similarly, in the study by Lim et al., it was seen that while parents said they wanted to be involved in pain care, they had a lack of adequate rest and nutrition, which limited their capacity to participate [4].

Social and cultural factors

Social and cultural factors was found to be a significant theme relating to mother participation in pain care and management. The culture and society of Saudi Arabia are rapidly changing, but patriarchy was still found to have a decisive influence on mother involvement. Rawas and Najjar [47] note that mothers traditionally have a limited role in decision-making regarding their child’s health care in Saudi Arabia, although women are becoming increasingly more empowered in this regard. In the present study, there was evidence that many still did not feel confident in making decisions and sometimes required a male family member to be present to determine pain care options. This is likely to change in future owing to changes in societal and health system policy, which advocate mothers' involvement, and also owing to changes in the general zeitgeist, which is moving toward an increasingly liberal outlook. However, empowering mothers in healthcare management is an area that requires addressing by healthcare systems. In addition, it was found that mothers’ cultural and religious beliefs sometimes hindered pain management, such as with respect to effective food waste disposal. Nurses recommended education programs for mothers with changes in healthcare policy that would permit a better understanding of pain management, in line with that practised in Western countries. An additional problem was that mothers were unable to take enough time off work to effectively care for their children, owing to employment commitments. More flexible working hours can give women more time to be involved with their children, which should be addressed by employers and employment agencies.

Hospital facilities, provisions, and services

Hospital facilities, provisions, and services was found to be a key theme connected to mother involvement. Many mother and nurse participants believed that entertainment, such as children’s toys, television, playrooms, and play therapists could be useful distractions for the child and lead to positive experiences, in line with recent research [48,49], and provides an extra point of support for mothers [3]. Children were observed to be frustrated owing to a lack of engagement in activities, and mothers and nurses expressed their dissatisfaction with the lack of provisions and services. This issue may be addressed by having 24-hour playroom facilities and regular visitation by play therapists, which have been used previously at the study hospital and could in principle be used in future. Indeed, playrooms and play therapists are particularly important for distraction, enabling greater social engagement with others, and improving psychological attitudes [50]. Mothers and nurses suggested using educational courses for nurses, counselling sessions for mothers, and group sessions for mothers and families, to improve interactions between patients and families and facilitate mother involvement. Twycross and Finley [9] and Shrestha-Ranjit and Manias [19] note that nurses often have insufficient knowledge regarding pain assessment and management in pediatric settings. Many nurses recommended the use of a dedicated pain management team in the pediatric surgical ward that would carry out routine visits. The team would manage pain treatment and provide information to mothers regarding the child’s condition and treatment, improving communication and understanding, and potentially improving mother involvement. This provision would follow the recommendation in Czarnecki et al.'s study, which posits that pain management teams could coordinate between parents, doctors, and nurses toward more favourable outcomes [18].

Recommendations

Health systems, institutions, and professionals must overcome obstacles to communication, in particular those related to the language barrier and internal communication between health professionals. Communication may be improved by providing Arabic language and culture courses for nurses, and employing cultural meditators and translators. Health systems, institutions, and professionals must consider the particular education and knowledge levels of mothers, as well as their preferences and cultural expectations, to enhance support. The use of entertainment provisions for children may enable a key distraction method to permit mothers to deal more successfully with hospitalization. Action must be taken to improve the emotional and physical support of mothers that work relatively independently in hospital settings. Familial support must be enhanced by exploring open visiting hours in pediatric departments. Since greater privacy is desired by mothers, hospital policy and provisions may consider using private rooms for mother-child dyads, especially in cases where mothers are highly involved in pain care and management, and when they are the main guardian or where there is little familial support. Nursing education programs can optimize nursing skills through the teaching of pain care setting for mothers and the use of analgesics. Pain management courses for mothers would enable greater confidence and skills in dealing with child pain care at the hospital and following discharge, and would lower hospital economic expenditure.

Limitations

This study included children with a variety of health issues, involving a variety of elective surgical treatments, meaning findings may be marginally inconsistent across the data sample since different procedures require varied input from parents. Also, since the majority of mother participants cared for boy child patients, this bias could have affected mother pain care involvement and management, because there are different attitudes and behaviours used with respect to caring for boy and girl patients.

In terms of health professional input, only the perspectives of nurses were included. However, doctors and other healthcare professionals have a decisive role in, and unique perspectives on, mothers' involvement, so their input should be incorporated into future research. Children’s own perspectives on mothers' involvement, and their self-reflexive perspectives, were not explored in this study, which might have led to greater insights into participation. Finally, since nurses were not assigned individually to child patients, but according to shift schedules,this might have affected results and could have been incorporated as a variable or control.

## Conclusions

The study has highlighted five main themes and a number of sub-themes that concern mothers' involvement in child postoperative pain care and management in a Saudi Arabian tertiary healthcare center. Health systems and health professionals in Saudi Arabia and beyond must inform and engage mothers, paying attention to required pain care knowledge, communication, physical and emotional support, social and cultural influences, and facilities and services to improve mother participation.
